# Metabolites Identification of Chemical Constituents From the Eggplant (*Solanum melongena* L.) Calyx in Rats by UPLC/ESI/qTOF-MS Analysis and Their Cytotoxic Activities

**DOI:** 10.3389/fphar.2021.655008

**Published:** 2021-07-15

**Authors:** Yuanyuan Song, Ting Mei, Yan Liu, Shengnan Kong, Jincheng Zhang, Minzhen Xie, Shan Ou, Meixia Liang, Qi Wang

**Affiliations:** ^1^ Department of Medicinal Chemistry and Natural Medicine Chemistry, College of Pharmacy, Harbin Medical University, Harbin, China; ^2^ Key Laboratory of Chinese Materia Medica, Heilongjiang University of Chinese Medicine, Harbin, China

**Keywords:** eggplant calyx, metabolite identification, amides, phenylpropanoids, cytotoxic activities

## Abstract

Eggplant (*Solanum melongena* L.) Calyx is a medicinal and edible traditional Chinese medicine with anti-inflammatory, anti-oxidant, and anti-cancer properties. However, the pharmacodynamic components and metabolic characteristics remain unclear. Amide and phenylpropanoid were the two main constituents, and four amides, including *n*-trans-*p*-coumaroyltyramine (**1**), *n*-trans-*p*-coumaroyloctopamine (**2**), *n*-trans-*p*-coumaroylnoradrenline (**3**), *n*-trans-feruloyloctopamine (**4**), and a phenylpropanoid neochlorogenic acid (**5**) were selected. In this study, these five representative compounds showed cytotoxic activities on A549, HCT116, and MCF7 cells. In addition, the metabolites of **1–5** from the eggplant calyx in rats were identified. In total, 23, 37, 29, and 17 metabolites were separately characterized in rat plasma, urine, feces, and livers, by UPLC/ESI/qTOF-MS analysis. The metabolism of amides and phenylpropanoid was mainly involved in hydroxylation, methylation, glucuronidation, or sulfation reactions. Two hydroxylated metabolites (**1-M2** and **2-M3**) were clearly identified by comparison with reference standards. Rat liver microsome incubation experiments indicated that P450 enzymes could hydroxylate **1–5**, and the methylation reaction of the 7-hydroxyl was also observed. This is the first study on the *in vivo* metabolism of these compounds, which lays a foundation for follow-up studies on pharmacodynamic evaluations and mechanisms.

## Introduction

*Solanum melongena* is the calyx of *Solanum melougeua* L., and it is a widely used traditional Chinese herb in medicinal and edible fields. It has been described as having anticancer, anti-inflammatory, antioxidant, and antiviral properties (Umamageswari and Maniyar, 2015; Di Sotto et al., 2018; Fekry et al., 2019; Jin et al., 2020; Khazaei et al., 2020). The chemical constituents of eggplant (*Solanum melongena* L.) mainly contain flavonoids, multiple alkaloids (including amides and glycoalkaloids), phenolic acids, and steroids. Among them, amides and phenolic acids are typically regarded as the primary and essential chemical components in eggplant (Meyer et al., 2015; Sun et al., 2015; Singh et al., 2017; Lelario et al., 2019). However, the metabolites and metabolic pathways of these components are not clear.

*Solanum melongena* extract has been frequently studied to treat several cancers, such as liver, cervical carcinoma, and breast cancer (Shabana et al., 2013). The *S. melongena* extract has significant beneficial effect on the treatment of Bowen’s disease. The extract could inhibit cell cycle progression in S-phase, inducing progressive cell apoptosis, and ultimately cell death (Sarah and misbahuddin, 2018). Interestingly, the many components of *Solanum melongena* exhibit multiple activities. Amides (*n*-trans-*p*-coumaroyltyramine, feruloyldopamine) have been shown to inhibit bacteria (Zacares et al., 2007). *N*-trans-coumaroyltyramine, *n*-trans-feruloyltyramine, and *n*-trans-feruloyloctopamine demonstrated effective radical scavenging activity (Li et al., 2012). The phenylpropanoid compound neochlorogenic acid revealed *in vitro* antioxidant behavior using three methods (ABTS, DPPH, and FRAP) (Zielinski et al., 2020). A variety of alkaloids can inhibit anticholinesterase activity related to the treatment of Alzheimer’s disease (Lelario et al., 2019). Here, we continued to investigate the cytotoxic activity of *Solanum Melougeua* L. compounds in other active sites. The alkaloids derived from *Solanum melongena* could protect Huh-7 and HepG2 liver cancer cells with IC50 values from 10.8 to 21.8 μM (Fekry et al., 2019). Moreover, *n*-trans-*p*-coumaroyloctopamine and *n*-trans-feruloyloctopamine also had antitumor activities against the human Caucasian prostate adenocarcinoma cell line PC-3, with IC50 values ranging from 69 to 99 μM (Happi et al., 2011). *N*-trans-feruloyloctopamine might occur directly through EMT-related signals (E-cadherin) and indirectly through the PI3K/Akt and p38 MAPK signaling pathways to inhibit cell invasion (Bai et al., 2017). Neochlorogenic acid exhibited an IC50 of 20 μM in human gastric carcinoma cells. Furthermore, it may inhibit the nude mouse xenograft model’s average tumour growth *in vivo* (Fang et al., 2019). However, most of these studies focus on the extract and lack of research on the principal component activity.

First, five main components were applied to A549 (human lung adenocarcinoma cell line), HepG2 (human liver cancer cell line), HCT116 (human colorectal cancer cell line), and MCF-7 (human breast cancer cell line), and the MTT assay was used to evaluate the cellular activities against lung cancer, liver cancer, rectal cancer, and breast cancer. On this basis, the metabolism of these active components was studied *in vivo*. Combined with previously published metabolic studies on *n*-trans-*p*-feruloyltyramine in *Datura metel* seeds, we characterized the metabolites of amides and phenylpropanoids, two primary components of the eggplant calyx in this study (Xu et al., 2018; Xia et al., 2019). A total of 37 metabolites were identified. We found that hydroxylation, methylation, glucuronidation, or sulfation were the main metabolic pathways of amides **1–4** and phenylpropanoid **5**. This study lays a solid foundation for follow-up studies on the cytotoxic activities of the main ingredients and the comprehensive identification of their metabolites in eggplant calyx.

## Materials and Methods

### Chemicals and Reagents

**FIGURE 1:**
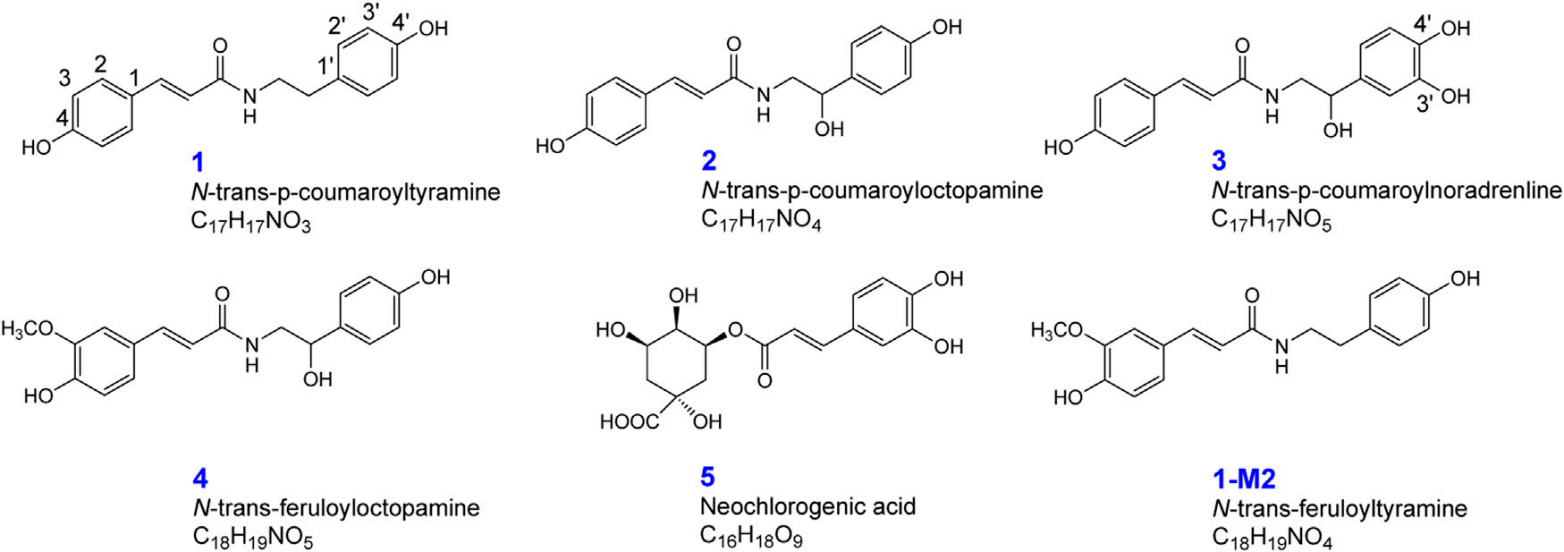
Chemical structures of *n*-trans-*p*-coumaroyltyramine (**1**), *n*-trans-*p*-coumaroyloctopamine (**2**), *n*-trans-*p*-coumaroylnoradrenline (**3**), *n*-trans-feruloyloctopamine (**4**), neochlorogenic acid (**5**), and *n*-trans-feruloyltyramine (**1-M2**).

The pure compounds *n*-trans-*p*-coumaroyltyramine **(1**), *n*-trans-*p*-coumaroyloctopamine **(2**), *n*-trans-*p*-coumaroylnoradrenline **(3**), *n*-trans-feruloyloctopamine **(4**), and neochlorogenic acid **(5**) were purchased from Nantong Feiyu Biological Technology Co., Ltd (Nanjing, China) by the authors. The metabolite **1-M2** (*n*-trans-*p*-feruloyltyramine) was also isolated from *Datura metel* seeds, most likely as artificial products. The five compound structures were distinguished by mass spectrometry, and the purities were above 98% according to HPLC/UV analysis (Figure 1). β-glucuronidase (100°KU) and rat liver microsomes were purchased from Sigma–Aldrich (St. Louis, MO, United States). Heparin was acquired from Beijing Xinyoubo Biological Technology Co., Ltd (Beijing, China). The other reagents were of analytical grade.

### Animals and Drug Administration

We bought male Sprague-Dawley rats (220∼250 g) from the Laboratory Animal Center of Second Affiliated Hospital of Harbin Medical University. The animal facilities and protocols were approved by the Animal Care and Use Committee of Harbin Medical University. All procedures were per the National Institutes of Health Guide for the Care and Use of Laboratory Animals (Institute of Laboratory Animal Resources, 1996). The rats were raised in metabolic cages (1,410 mm × 400 mm × 1,550 mm) and had free access to water and normal chow *ad libitum* on a 12 h dark-light cycle for 3 days. The breeding room temperature and humidity was 25°C and 60 ± 5%, respectively. All rats fasted 12 h before experiments. The five monomer compounds were separately suspended in 1% carboxy-methyl cellulose sodium to obtain solutions (2 mg/ml for each compound). The solutions were orally administered to rats (*n* = 2) at 20 mg/kg, and the control was given 2 ml normal saline.

Rats in different groups took the drug, and blood was collected in heparinized tubes from the angular vein at 0.5, 1, 2, 4, 6, and 8 h after administering each pure compound (two rats for each time point). The urine and feces composition was collected and uncovered to observe which components were metabolized by the drugs.

### Preparation of Plasma, Urine, and Fecal Samples

For each rat, the blood was centrifuged at 6,000 rpm for 10 min to obtain plasma. Upon merging plasma of each time point, 3 ml plasma was treated with four volumes of methanol and acetonitrile to precipitate protein. The mixture was vortexed at 2,200 rpm for 5 min and centrifuged at 9,000 rpm for 10 min. The supernatant was separated and dried with nitrogen at 37°C. The residue was dissolved in 300 μl of methanol and filtered through a 0.22 μm membrane for Triple TOF^®^ 5600 + LC/MS/MS analysis.

The rats were placed in metabolic cages, and urine and fecal samples were collected within 24 h after administration. The urine samples were treated and purified by a methanol-activated solid-phase extraction column (Oasis HLB 6 cc). After activating the column, 4 ml urine samples were successively eluted with 5 ml of water, 5 ml of 5% methanol, and 5 ml of methanol. The methanol eluate was collected and evaporated to dryness in a water bath at 37°C under reduced pressure. The residue was redissolved in 300 μl methanol, filtered through a 0.22 μm filter membrane, and analyzed by Triple TOF^®^ 5600 + LC/MS/MS.

Feces were amassed from the rats within 24 h after administration, dried in air, and ground into powder. Powder (1.0 g) was extracted with 20 ml methanol in an ultrasonic bath for 30 min. After extraction, the supernatant was centrifuged, evaporated to dryness under reduced pressure at 37°C, dissolved in 200 μl methanol, and filtered with a 0.22 μm filter membrane before analysis.

### Preparation of Liver Tissue Samples

After liver tissue homogenization, the liver tissue (0.1 g) was treated by ultrasonication with 4 ml of methanol for 30 min as the extraction condition. Then, the supernatant was centrifuged at 9,000 rpm for 10 min. Next, the supernatant was allowed to dry with nitrogen, redissolved with 300 µl methanol, and filtered through a 0.22 μm membrane.

### Incubation of Rat Liver Microsomes

Compounds **1–5** were separately dissolved in methanol, and then used phosphate buffered saline (PBS) to dilute. 200 µl incubation mixture (NADPH-generating system, 100 mM potassium phosphate buffer (pH 7.4), and rat liver microsomes were mixed, and its final concentration of each compound was 25 µM. The amount of organic solvent in the mixture was lower than 1% (v/v). PBS-containing methanol was used as a negative control. The incubation was conducted at 37°C for 2 h and was terminated by adding 1,000 µl of cold acetonitrile. It needed to keep the mixture was at 4°C for 30 min and removed the precipitated protein by centrifugation at 10,000 × *g* for 10 min at 4°C.

### UPLC/ESI/qTOF-MS Analysis

The ACQUITY UPLC system (Waters, United States) equipped with an ESI ion source operating in both positive and negative ion mode and an autosampler were controlled with MassLynx^™^ (V4.1) software. An AB SCIEX Triple TOF 5600 was used for chromatographic separation. An ACQUITYUPLC CSH^™^ Phenyl-Hexyl (2.1 mm × 100 mm, 1.7 μm; Waters) was utilized for chromatographic separation. The mobile phase consisted of water A) containing 0.1% (v/v) formic acid and acetonitrile B) at a flow rate of 0.4 ml/min. The pressure limit is 15,000°psi. The gradient elution program was set as follows: initial, 95% A, 5% B; 8°min, 5% A, 95% B; 9°min, 95% A, 5% B; 10°min, 95% A, 5% B. A 10 μl sample aliquot was injected onto the column, with the column temperature maintained at 35°C.

According to our optimized conditions, the product ion system was equipped with an ESI source operating in positive ion mode. The MS full scan range was 150–1,200°m/*z*, and the product ion scan range was 80–1,000°m/*z*. The optimized parameters were as follows: capillary voltage, 5.5 kV; declustering potential, 80 V; collision energy, 35 V. High-purity nitrogen (N_2_) and high-purity argon (Ar) were separately used as the desolvation and collision gas, respectively. The flow rate of cone gas (N_2_) was 0.8 l/min. The desolvation and source temperatures were 450 and 100°C, respectively. All data obtained in positive ion mode was acquired and processed by MassLynx^™^ (V4.1) software.

### β-Glucuronidase Hydrolyzation

The plasma or urine need to been dried under nitrogen gas. Then mixed the 100 µl sample with 400 µl of β-glucuronidase solution (containing 19.86 U/µl, in sodium acetate buffer, pH 5.5), vortexed for 5 min, incubated in a 37°C water bath for 1.5 h, treated with 1,000 µl of cold methanol–acetonitrile (1:1, v/v) to precipitate the protein, and centrifuged at 10,000 × *g* for 10 min at 4°C. Finally, the supernatant should be dried under a gentle stream of nitrogen and then dissolved in 100 µl of methanol. The solution was filtered through a 0.22 µm membrane for analysis.

### Cytotoxic Activity Assay

Cytotoxic activities of compounds **1–5** against A549 (human lung adenocarcinoma cell), HepG2 (human liver carcinoma cell line), HCT116 (human colorectal cancer cell), and MCF7 (human breast carcinoma cell line) cells were determined using the MTT assay. HepG2 and MCF7 cells were attained from ATCC and cultured at 37°C in a humidified 5% CO_2_ atmosphere in DMEM (A549 cells in F-12 K medium and HCT116 cells in McCOY’s 5 A medium) supplemented with 10% fetal bovine serum and 1× penicillin-streptomycin solution. The cells were briefly seeded at 5 × 10^3^ cells/well in 96-well plates and cultured overnight. According to the results of the previous multi-concentration screening of the same type of compounds in varying concentrations, compounds **1–5** were then added to the culture in 10 μM and incubated for another 24, 48, and 72 h. The MTT assay measured cell availability following the manufacturer’s protocol (Promega, Madison, WI, United States). Irinotecan was used as the positive control.

## Results and Discussion

### Cytotoxic Activities of Eggplant Calyx Compounds **1–5** Against A549, HepG2, HCT116 and MCF7 Cells

**FIGURE 2:**
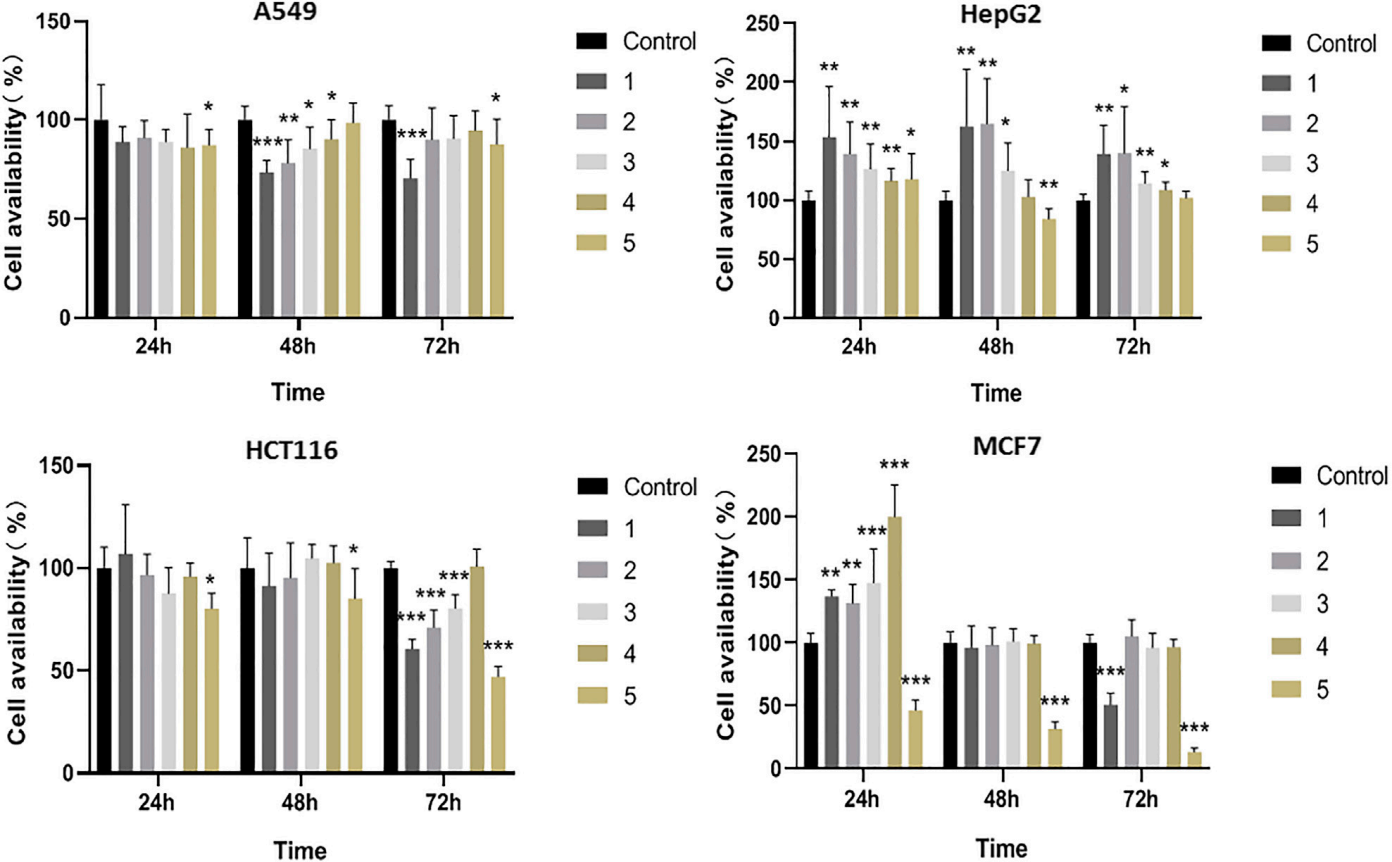
The cytotoxic effects of test compounds **1–5** against A549, HepG2, HCT116 and MCF7 cells.

Eggplant calyx and its compositions have been reported to possess remarkable antitumor behaviors (Fekry et al., 2019; Jin et al., 2020). Most research concentrates on the antioxidant and antibacterial activities of these five compounds, while the anticancer actions of **2** and **5** are studied on a smaller scale (Happi et al., 2011; Fang et al., 2019). In this study, the cytotoxicities of eggplant calyx compounds (10 μM) against four human cancer cell lines, namely, A549, HepG2, HCT116, and MCF7, were evaluated. Their cytotoxic actions against these cell lines were tested by MTT assay. Amides **1**–**4** and phenylpropanoid **5** showed cytotoxic activities, as presented in Figure 2.

Phenylpropanoid **5** displayed evident cytotoxic performance against all four cell lines. Especially for MCF7 cells, the inhibition rate reached 86.5% at 10 μM. Compounds **1–4** showed no significant change in cytotoxic activity over time in different cell lines, while the inhibition effect of **5** in MCF7 cells gradually increased with time, and the inhibition rate reached 86.5% at 72 h. Moreover, amide **1** inhibited A549, HCT116, and MCF7 cell availability by 29.6, 39.4, and 49.2% at 10 μM, respectively. **2** and **3** only exhibited inhibitory effects on A549 and HCT116 cells, while **4** revealed no significant cytotoxic activity (Figure 2; Supplementary Tables S1–S4). In addition, the compounds could promote cell proliferation in HepG2 cells. It was possible that these compounds had different targets on HepG2 cells and other tumor cells, and therefore have different effects.

### Metabolites Identification of **1**–**5**

These five bioactive compounds from the eggplant calyx were selected to identify their metabolites in rats (Figure 1). The plasma, urine, fecal, and liver tissue samples were rapidly analyzed using a TripleTOF^®^ 5,600 + LC/MS/MS method to describe their metabolites in supplementing their fragmentation (Table 1; Supplementary Figures S4–S8). Phase I metabolites of **1–5** were confirmed by liver tissue and rat liver microsome incubation samples. Meanwhile, phase II metabolites were confirmed by β-glucuronidase hydrolysis. The structures of metabolites **1-M2** and **2-M3** were identified by comparison with reference standards.

**TABLE 1:** Characterization of *in vivo* metabolites of eggplant green calyx compounds **1**–**5** by HPLC/ESI-IT-TOF-MS.

No	RT (min)	Formula	HR-MS [M + H]^+^			(+)ESI-MS^n^(*m*/*z*)	Metabolic reaction	Plasma	Urine	Feces	Liver
			Measured	Predicted	Δ (ppm)						
**1** ^a^	6.50	C_17_H_17_NO_3_	284.3278	284.3291	−4.5	MS^2^ [284]: 163,147,137,106	*N*-trans-*p*-coumaroyltyramine	−	++	++	+
** 1-M1**	4.26	C_17_H_17_NO_4_	300.3278	300.3285	−2.3	MS^2^ [300]: 163,137	+OH	−	+	++	+
** 1-M2** ^a^	5.65	C_18_H_19_NO_4_	314.3522	314.3551	−9.2	MS^2^ [314]: 177,163,137	+OH + CH_3_ (*N*-trans-*p*-feruloyltyramine)	−	+	+	+
** 1-M3**	5.71	C_17_H_17_NO_6_S	364.3910	364.3923	−3.5	MS^2^ [364]: 284,147	+Sul	+	+	+	−
** 1-M4**	5.78	C_17_H_17_NO_7_S	380.3954	380.3917	9.7	MS^2^ [380]: 342,300,163	+Sul + OH	−	+	+	−
** 1-M5** ^b^	5.96	C_23_H_25_NO_9_	460.4543	460.4532	2.3	MS^2^ [460]: 284	+GluA	+	+	++	−
						MS^3^ [284]: 163,147,137					
** 1-M6** ^b^	6.08	C_23_H_25_NO_12_S	540.5098	540.5164	6.2	MS^2^ [540]: 364	+Sul + GluA	−	+	+	−
						MS^3^[364]: 284,215,147,137					
**2** ^a^	5.88	C_17_H_17_NO_4_	300.3262	300.3285	−7.6	MS^2^ [300]: 153,147	*N*-trans-*p*-coumaroyloctopamine	+	+	++	+
** 2-M1**	2.63	C_17_H_17_NO_5_	316.3291	316.3279	3.7	MS^2^ [316]: 163,152	+OH	+	++	++	+
** 2-M2**	3.13	C_17_H_15_NO_3_	282.3129	282.3132	−1.0	MS^2^ [282]: 163,135	-H_2_O	−	++	+	−
** 2-M3** ^a^	5.41	C_18_H_19_NO_5_	330.3540	330.3545	−1.5	MS^2^ [330]: 177, 152	+OH + CH_3_ (‡4)	+	+	−	+
** 2-M4**	5.50	C_17_H_17_NO_8_S	396.3928	396.3911	4.2	MS^2^ [396]: 316	+Sul + OH	+	+	+	−
						MS^3^[316]: 163,122					
** 2-M5** ^b^	5.58	C_23_H_25_NO_10_	476.4500	476.4526	−5.4	MS^2^ [476]: 300, 163,153	+GluA	+	++	−	−
** 2-M6** ^b^	5.63	C_23_H_25_NO_13_S	556.5148	556.5158	−1.7	MS^2^ [556]: 380	+Sul + GluA	−	+	+	−
						MS^3^[380]: 300, 147					
**3** ^a^	8.83	C_17_H_17_NO_5_	316.3250	316.3279	−9.1	MS^2^ [316]: 168,147	*N*-trans-*p*-coumaroylnoradrenline	+	++	+	+
** 3-M1**	2.01	C_17_H_17_NO_6_	332.3252	332.3273	−6.3	MS^2^ [332]: 168,163,112	+OH	++	+	+	++
** 3-M2**	3.41	C_17_H_15_NO_4_	298.3120	298.3126	−2.0	MS^2^ [298]: 150,147	-H_2_O	−	+	+	−
** 3-M3**	5.12	C_18_H_19_NO_6_	346.3530	346.3539	−2.5	MS^2^ [346]: 177,168,121	+OH + CH_3_	++	++	−	+
** 3-M4**	6.09	C_17_H_17_NO_8_S	396.3933	396.3911	5.5	MS^2^ [396]: 316,168,163,149	+Sul	+	+	+	−
** 3-M5**	7.79	C_17_H_17_NO_9_S	412.3915	412.3905	2.4	MS^2^ [412]: 332,219,163	+Sul + OH	+	+	−	−
** 3-M6** ^b^	8.25	C_23_H_25_NO_11_	492.4544	492.4520	4.8	MS^2^ [492]: 316	+GluA	++	+	−	−
						MS^3^[316]: 168,147					
** 3-M7** ^b^	8.72	C_23_H_25_NO_14_S	572.5129	572.5152	−4.0	MS^2^ [572]: 316	+Sul + GluA	++	+	+	−
						MS^3^[316]: 147					
** 3-M8**	8.93	C_17_H_15_NO_5_	314.3119	314.3120	−3.1	MS^2^ [314]: 166,147,121	-2H	+	+	−	+
**4** ^a^	5.81	C_18_H_19_NO_5_	330.3536	330.3545	−2.7	MS^2^ [330]: 177, 152,121	*N*-trans-feruloyloctopamine	++	++	+	++
** 4-M1**	3.55	C_18_H_19_NO_6_	346.3511	346.3539	−8.0	MS^2^ [346]: 177,168	+OH	+	+	+	+
** 4-M2**	4.55	C_19_H_21_NO_5_	344.3827	344.3811	4.6	MS^2^ [344]: 226,191,177,152	+CH_3_	++	+	−	+
** 4-M3**	5.15	C_18_H_19_NO_9_S	426.4170	426.4171	−0.2	MS^2^ [426]: 346	+Sul + OH	−	+	−	−
						MS^3^ [346]:177,168,152					
** 4-M4** ^b^	5.63	C_24_H_27_NO_11_	506.4762	506.4786	−4.7	MS^2^ [506]: 330	+GluA	++	+	+	−
						MS^3^[330]: 177, 121					
** 4-M5** ^b^	5.65	C_24_H_25_NO_11_	504.4621	504.4627	−1.1	MS^2^ [504]: 328	+GluA-2H	+	+	+	−
						MS^3^[328]: 177					
** 4-M6** ^b^	5.69	C_24_H_27_NO_14_S	586.5435	586.5418	2.8	MS^2^ [586]: 320	+Sul + GluA	−	+	−	−
						MS^3^ [320]: 152,136					
**5** ^a^	9.46	C_16_H_18_O_9_	355.3167	355.3161	1.6	MS^2^ [355]: 313	Neochlorogenic acid	−	+	++	+
						MS^3^ [313]: 191,179,175,163,138					
** 5-M1**	3.25	C_16_H_16_O_8_	337.3030	337.3008	6.5	MS^2^ [337]: 179,163	−H_2_O	−	++	++	−
** 5-M2**	4.33	C_17_H_20_O_10_	385.3410	385.3420	−2.5	MS^2^ [385]:355, 191,158	+OH + CH_3_	+	+	+	+
** 5-M3**	4.39	C_17_H_20_O_9_	369.3451	369.3426	6.7	MS^2^ [369]: 355,191,163,137	+CH_3_	+	+	++	+
** 5-M4**	6.40	C_16_H_18_O_10_	371.3152	371.3155	−0.8	MS^2^ [371]: 355	+OH	−	++	++	+
						MS^3^ [355]:191					
** 5-M5**	8.08	C_16_H_18_O_12_S	435.3799	435.3793	1.3	MS^2^ [435]: 355	+Sul	+	+	+	−
						MS^3^ [435]: 355,191,179,163					
** 5-M6**	8.16	C_16_H_18_O_13_S	451.3791	451.3787	0.8	MS^2^ [451]: 381,163	+Sul + OH	−	+	++	−

++, detected at high abundance; +, detected; −, not detected.

a Identified by comparing with reference standards.

b Confirmed by enzyme hydrolysis.

#### Metabolite Identification of *n*-Trans-*p*-Coumaroyltyramine (**1**)

**FIGURE 3:**
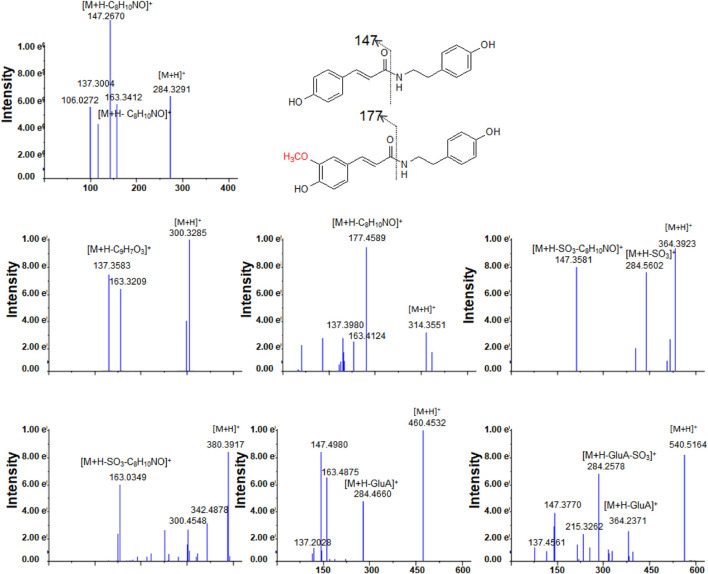
The tandem mass spectra for **1** and its metabolites.

**FIGURE 4:**
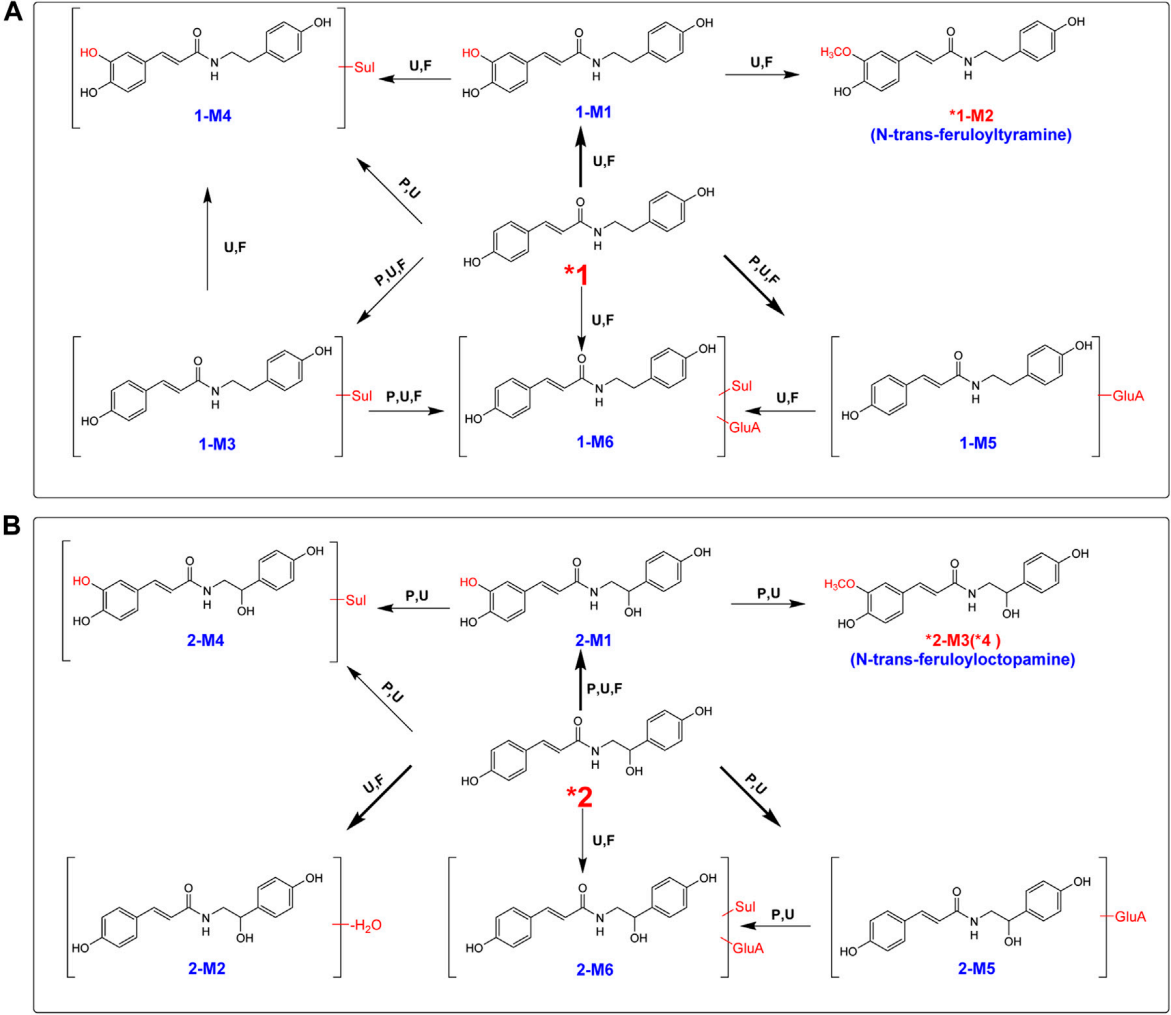
Proposed metabolic pathway for **1 (A)** and **2 (B)** in rats after oral administration. Bold red arrows indicate major metabolites; *, compared with reference standards; U, detected in urine; *p*, detected in plasma; F, detected in feces; RLM, detected in rat liver microsomes; Sul, sulfate; GluA, glucuronic acid residue.

After oral administration of a 20 mg/kg dose, **1** could be detected in rat urine, fecal, and liver tissue samples by LC/MS in negative or positive ionization mode. Nevertheless, it could not be identified in plasma. **1** mainly underwent hydroxylation (**1-M1**) and glucuronidation (**1-M5**) reactions (Table 1; Supplementary Figure S4). Methylation after hydroxylation (**1-M2**) and sulfation and hydroxylation (**1-M4**) were observed (Figure 3). Moreover, **1** could also produce sulfate conjugates (**1-M3**); sulfated and glucuronidated metabolites (**1-M6**) were also discovered. The detailed metabolic pathways of **1** are proposed in Figure 4A. **1-M3** and **1-M5** were distributed in plasma, urine and feces; **1-M1** and **1-M2** were distributed in urine, feces and liver; **1-M4** and **1-M6** were only distributed in urine and feces (Table 1).

High-resolution mass spectra of **1-M1** confirmed an [M + H]^+^ ion at *m*/*z* 300.3278. Its molecular formula was established as C_17_H_17_NO_4_, which was a hydroxylated metabolite of **1**. Its MS/MS spectrum displayed fragment ions at *m*/*z* 163 [(M + H-C_8_H_10_NO)^+^], 147 [(M + H-C_8_H_10_NO)^+^], and 137 [(M + H-C_9_H_7_O_3_)^+^], generated from parent compound **1** due to the break of the peptide bond (Lin et al., 2015; Conic et al., 2020) (Figure 3).

Methylation after hydroxylation product **1-M2** was detected in rat urine, feces, and liver tissue samples, and the HRESIMS spectra presented the molecular formula of **1-M2** as C_18_H_19_NO_4_. By comparison with a reference standard, **1-M2** was clearly identified as *n*-trans-*p*-feruloyltyramine, isolated from the seeds of eggplant calyx, and the methylated reaction was substituted at the C-3 hydroxyl group [19] (Supplementary Figure S1). Moreover, **1-M2** was also distinguished by a rat liver microsome incubation experiment, demonstrating that P450 enzymes catalyzed the hydroxylation reaction in the liver (Supplementary Figures S1, S3).

The high-resolution mass spectra ascertained the molecular formula of **1-M3** as C_17_H_17_NO_6_S (*m*/*z* 364). In tandem mass spectra, **1-M3** produced fragment ions *m*/*z* 284 [(M + H-SO_3_)^+^] and *m*/*z* 147 [(M + H-SO_3_-C_8_H_10_NO)^+^], indicating that it was a sulfated conjugate. The HRESIMS spectra showed the molecular formula of **1-M4** as C_17_H_17_NO_7_S, signifying it was a sulfated and hydroxylated conjugate of **1**. The hydroxyl group might occur at C-3, according to the fragment ions *m*/*z* 163 [(M + H-SO_3_-C_8_H_10_NO)^+^]. The glucuronidated conjugate **1-M5** was observed in plasma, urine, and feces in high abundance. Its tandem mass spectra were dominated by the neutral loss of 176 Da (glucuronic acid residue). The high-resolution mass spectra of **1-M6** showed an [M + H]^+^ ion at *m*/*z* 540.5098, representing the molecular formula of C_23_H_25_NO_12_S. It was the sulfated and glucuronidated product. The sulfation and glucuronidation positions could not be assigned due to limited structural information (Figures 3, 4A).

#### Metabolite Identification of *n*-Trans-*p*-Coumaroyloctopamine (**2**)

Contrary to **1**, **2** occurred in the unchanged form in rat plasma, urine, and feces in a large portion at a dosage of 20 mg/kg, and we observed that a small portion of **2** was metabolized (Table 1; Supplementary Figure S5). Four metabolites were detected in plasma, including hydroxylated (**2-M1**), hydroxylated and methylated (**2-M3**), hydroxylated and sulfated (**2-M4**), and glucuronidation (**2-M5**) (Table 1; Figure 4B). Hydroxylation and glucuronidation were the major metabolic reactions. Similar to **1-M1** and **1-M2**, **2-M1** and **2-M3** were also identified in rat liver tissue and microsomes, signifying that P450 enzymes catalyzed the hydroxylation and methylation reactions.

**2-M3** was methylated at the hydroxyl group at C-3. The retention time of metabolite **2-M3** was 5.81 min, which was consistent with that of compound **4**. Meanwhile, upon collision-induced dissociation, the [M + H]^+^ ion could fragment into *m*/*z* 177 [(M + H-C_8_H_10_NO_2_)^+^] and 152 [(M + H-C_10_H_9_O_3_)^+^], which were also generated from **4**. Therefore, the structure of **2-M3** was identified as *n*-trans-feruloyloctopamine.

**FIGURE 5:**
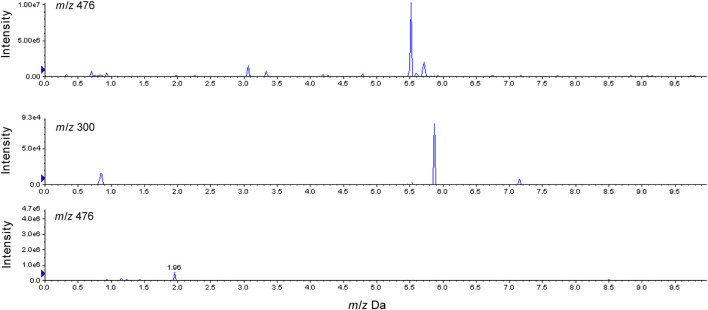
Characterization of glucuronide conjugates in rats urine after oral administration of **2** before and after β-glucuronidase hydrolysis.

The high-resolution mass spectrum of **2-M1** confirmed the molecular formula C_17_H_17_NO_5_, signifying a monohydroxylated derivative of **2**. Its MS/MS spectrum revealed fragment ions at *m*/*z* 163 [(M + H-C_8_H_10_NO_2_)^+^] and 152 [(M + H-C_9_H_7_O_3_)^+^], and the hydroxylation reaction may take place at C-3, similar to **1-M1**. **2-M2** was a dehydration derivative of **2**. Its MS/MS spectrum displayed fragment ions at *m*/*z* 135 [(M + H-H_2_O-C_9_H_7_O_2_)^+^]. Three phase II metabolites of **2** were discovered in rat plasma, urine, and feces, including sulfation (**2-M4**) and glucuronidation (**2-M5**), and their conjugates (**2-M6**). The peaks disappeared when the urine samples were treated with β-glucuronidase (Figure 5).

#### Metabolite Identification of *n*-Trans-*p*-Coumaroylnoradrenline (**3**)

**FIGURE 6:**
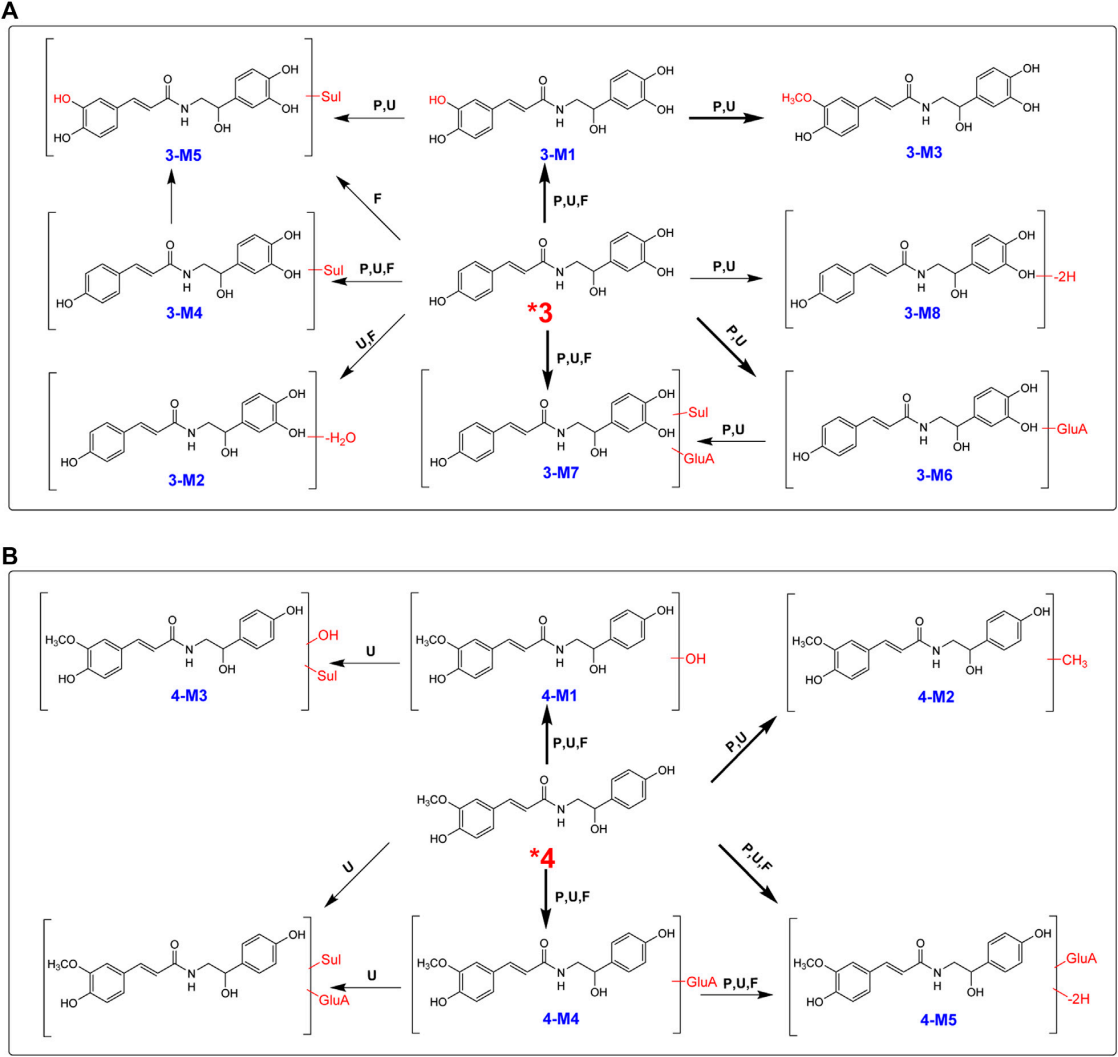
Proposed metabolic pathways for **3 (A)** and **4 (B)** in rats after oral administration.

After oral administration, a large portion of **3** was metabolized, and eight metabolites were found in rat plasma, urine, and feces in high abundance (Table 1; Figure 6A). Hydroxylation, hydroxylation and methylation, and glucuronidation were the key metabolic reactions for **3**. All the eight metabolites were found in urine. Except 3-M2, the other seven metabolites were found in plasma.

High-resolution mass spectra of **3-M1** gave [M + H]^+^ signals at *m*/*z* 332.3252, and the molecular formula was established as C_17_H_17_NO_6_, implying that it was a hydroxylated derivative. The fragmentation pattern for **3-M2** was very similar to the metabolism of **2**, suggesting that hydroxylation may be substituted at C-3. The molecular formula of **3-M3** was reasoned to be C_18_H_19_NO_6_ by high-resolution mass spectral analysis, suggesting a methylated and hydroxylated product of **3**.

In total, four phase II metabolites (**3-M4**, **3-M5**, **3-M6**, and **3-M7**) were present in rat plasma and urine after oral administration of **4** (Supplementary Figure S6). The peaks would disappear after enzyme hydrolysis. Their MS/MS spectra were dominated by the neutral loss of 80 Da (sulfate residue) or 176 Da (glucuronic acid residue).

#### Metabolite Identification of *n*-Trans-Feruloyloctopamine (**4**)

After an oral dose of 20 mg/kg, **4** could be readily absorbed into circulation and occurred mainly as the unchanged form in plasma, urine, and liver tissue, together with six metabolites (Supplementary Figure S7). Three glucuronic acid-conjugated phase II metabolites (**4-M4**, **4-M5**, and **4-M6**) were uncovered in plasma, urine, and feces. The neutral loss of 176 Da dominated the MS/MS spectra of **4**-**M4**. **4-M5** [(M + H]^+^ *m*/*z* 504.4621, C_24_H_25_NO_11_)] was a dehydrogenated derivative of **4-M4**. According to the high-resolution mass spectra, **4-M6** was characterized as **4**-*O*-glucuronide-*O*-sulfate [C_24_H_27_NO_14_S (M + H)^+^ *m*/*z* 586.5435] (Figure 6B).

The high-resolution mass spectra of metabolite **4-M1** exhibited an [M + H]^+^ ion at *m*/*z* 346.3511, representing the molecular formula of C_18_H_19_NO_6_, which is a hydroxylated metabolite and was examined in plasma, urine, and feces. The MS/MS spectrum showed that the [M + H]^+^ ion produced fragment ions at *m*/*z* 177 [(M + H-C_8_H_10_NO_3_)^+^] and *m*/*z* 168 [(M + H-C_10_H_9_O_3_)^+^] (Figure 6B). **4-M3** [C_18_H_19_NO_9_S (M + H)^+^ *m*/*z* 426.4170] was described as sulfation of **4-M1**. **4** could add the methyl group to produce **4-M2** [C_19_H_21_NO_5_ (M + H)^+^ *m*/*z* 344.3827], and the chief metabolic reaction was methylation.

#### Metabolites Identification of Neochlorogenic Acid (**5**)

The metabolism of phenylpropanoid **5** is entirely different from that of amides. A total of four phase II metabolites were identified in rat plasma after oral administration of **5**. It mostly undergoes dehydration, hydroxylation, methylation, in addition to sulfation and hydroxylation reactions. A total of six metabolites were characterized in both urine and feces (Supplementary Figure S8). The molecular formula of **5-M1** was inferred to be C_16_H_16_O_8_ by high-resolution mass spectral analysis, denoting a dehydrated product of **5**. The MS/MS spectrum proved that the [M + H]^+^ ion produced fragment ions at *m*/*z* 179 (C_9_H_7_O_4_•, caffeic acid). Upon collision-induced dissociation of **5-M2**, the [M + H]^+^ ion at *m*/*z* 385 could produce *m*/*z* 191 (C_7_H_11_O_6_•) due to cleavage of quinic acid (Supplementary Figure S2). A close analog, chlorogenic acid, had similar metabolic patterns (Olthof et al., 2003). **5-M5** was a sulfate of **5** [C_16_H_18_O_12_S (M + H)^+^ *m*/*z* 435], and **5-M6** was a sulfate of hydroxylated **5** [C_16_H_18_O_13_S (M + H)^+^ *m*/*z* 451]. **5-M3** [C_17_H_20_O_9_ (M + H)^+^ *m*/*z* 369] and **5-M4** [C_16_H_18_O_10_ (M + H)^+^ *m*/*z* 371] were regarded as methylated and hydroxylated metabolites of **5**, respectively. The high-resolution mass spectrum of **5-M2** established its molecular formula as C_17_H_20_O_10_, demonstrating a methylated and hydroxylated product of **5**. There were only two phase II metabolites, and **5-M2** was detected in the plasma sample. Supplementary Figure S2 proposes a metabolic pathway for **5**.

### Metabolic Pathways of the Compounds in Eggplant Calyx

The metabolism of five bioactive compounds of eggplant calyx representing significant structural types in rats was studied. Amides **2, 3,** and **4** were easily absorbed into circulation and occurred predominantly in the unchanged form in both plasma and urine. However, **1** showed poor oral absorption, and most of their metabolites were only discovered in urine and feces. This may be due to the increase in hydroxyl groups, and the content of amide prototype components in the blood gradually increased (Choppala et al., 2013; Ouiyangkul et al., 2020). Among the phase I metabolites, hydroxylated and methylated products could be observed in plasma at relatively high amounts, consistent with our previous findings (Xu et al., 2018; Xia et al., 2019). The hydroxylation and methylation reactions for **1**, **2**, and **3** took place at C-3 and hydroxyl groups at C-3, respectively. Glucuronidation was also the primary metabolic reaction for amides. Hydroxylated products were detected in the liver and *in vitro* liver microsome-incubated samples. Hydroxylation may occur mainly due to the role of metabolic enzymes in the liver, such as P450 2D2 and P450 2C9 (Hosoi et al., 2012; Uehara et al., 2019). The causes of methylation include genetic polymorphisms in cytochrome P450 metabolizing enzymes and polymorphisms in amine methyltransferase enzymes. However, the mechanism by which methylation occurs is unclear and warrants further investigation (Kurpius and Alexander, 2006; Manikandan and Nagini, 2018).

We noticed neochlorogenic acid **(5**) exhibited significant cytotoxic activity against the A549, HepG2, HCT116, and MCF-7 cell lines, and the inhibition rates were 12.3, 15.7, 53.0, and 86.5%, respectively. The results were similar to those of chlorogenic acid, a similar-structure chemical. Some previous studies have reported the antiproliferative and cytotoxic activity of chlorogenic acid against human breast (MDAMB-231 and MCF-7), lung (A549), colon (HCT116 and HT29), bone (MG-63 and Saos-2), and kidney cancer cells (A498 and HEK293) (Mohammed et al., 2016; Santana-Galvez et al., 2020). Moreover, **5** could generally undergo dehydration, methylation, hydroxylation, and sulfation plus hydroxylation; metabolites were detected in urine and feces. Interestingly, the metabolites of **5** were more abundant in urine and feces than the prototype, signifying the human body could extensively metabolize **5**. This may be due to the presence of ester bonds between caffeic and quinic acid.

The five bioactive constituents **2–4** in eggplant calyx mostly displayed excellent oral bioavailability, which was found in plasma, urine, and liver tissues in notable amounts. However, **1** and **5** were not detected in plasma. All these constituents both undergo hydroxylation, methylation, glucuronidation, and sulfation reactions, which have not yet been reported for the constituents from eggplant (*Solanum melongena* L.) calyx in rats. The presence of hydroxyl groups (**1**, **2**, **3**, **4**, **5**), methoxy groups **(4**), and ester bonds **(5**) may affect their oral bioavailability. Although **2**, **3,** and **4** both contain 4-OH and 4′-OH, **4** showed extraordinarily higher oral absorption than **2** and **3**, perhaps due to the existence of 3-OCH_3_.

## Conclusion

The metabolism of five representative bioactive compounds **1–5** of eggplant calyx after oral administration to rats was studied, and the majority could be readily absorbed into circulation. **1–5** exhibited inhibitory effects on A549, HCT116, and MCF7 cells. It is worth noting that phenylpropanoid **5** and amide **1** showed prominent cytotoxic activities, and the inhibition rates were 86.5 and 49.2% in MCF7 cells at 10 μM, respectively. Additionally, 32 metabolites were characterized in rat biological samples by UPLC/ESI/qTOF-MS analysis and β-glucuronidase hydrolysis. The metabolism of amides **1–4** mainly involves hydroxylation, hydroxylation and methylation, glucuronidation, or sulfation reactions. Phenylpropanoid **5** could undergo hydroxylation, methylation, hydroxylation and methylation, dehydration, or sulfation reactions. To our knowledge, this is the first study on the *in vivo* metabolism of these five bioactive constituents. These results are valuable in evaluating cytotoxic activities and predicting the metabolism of other amide and phenylpropanoid compounds with similar structures from *Solanum melongena* L. The findings may promote systematic multicomponent metabolism and the clinical therapeutic effects of eggplant calyx.

## Data Availability

The original contributions presented in the study are included in the article/Supplementary Material, further inquiries can be directed to the corresponding author.
