# Supplementary material for: Metabolites Identification of Chemical Constituents From the Eggplant (Solanum melongena L.) Calyx in Rats by UPLC/ESI/qTOF-MS Analysis and Their Cytotoxic Activities
Source: Front Pharmacol. 2021 Jul 15;12:655008. doi: 10.3389/fphar.2021.655008 (PMC8320773; doi:10.3389/fphar.2021.655008)
Supplement: Supplementary file 1 [file Presentation1.pdf]

Metabolites identification of chemical constituents from the eggplant (*Solanum melongena* L.) calyx in rats by UPLC/ESI/qTOF-MS analysis and their cytotoxic activities

Yuanyuan Song [a,1], Ting Mei [a,1], Yan Liu [b,1], Shengnan Kong [b], Jincheng Zhang [a], Minzhen Xie [a], Shan Ou [a], Meixia Liang [a], Qi Wang [a,*]

Affiliation:

[a] *Department of Medicinal Chemistry and Natural Medicine Chemistry, College of Pharmacy, Harbin Medical University, Harbin 150081, China*

[b] *Key Laboratory of Chinese Materia Medica, Heilongjiang University of Chinese Medicine, Harbin 150040, China*

***Correspondence:**

Qi Wang, PhD, Department of Medicinal Chemistry and Natural Medicine Chemistry, Harbin Medical University, 157 Baojian Road, Nangang District, Harbin, Heilongjiang 150081, China. Tel: 86-451-86660227, E-mail address: mydearmumu@163.com.

[1]. These authors contributed equally to this work.

Table of Contents

Fig. S1. Base peak chromatograms of **1** in rats liver after oral administration (A), extracted ion chromatograms of **1-M2** in rat liver microsomes (B), and the standard solution of **1-M2** (C) .

Fig. S2. Proposed metabolic pathway for **5** in rats after oral administration.

Fig. S3. TIC chromatograms of metabolites of *n*-trans-p-coumaroyltyramine (**1**, a), *n*-trans-p-coumaroyloctopamine (**2**, b), *n*-trans-p-coumaroylnoradrenline (**3**, c), *n*-trans-feruloyloctopamine (**4**, d), phenylpropanoid neochlorogenic acid (**5**, e) in rat liver microsomes in vitro.

Fig. S4. Total ion chromatograms (a) and extracted ion chromatograms (b, c, d, e, f, g, h) of compound **1** and its metabolites in rat urine.

Fig. S5. Total ion chromatograms (a) and extracted ion chromatograms (b, c, d, e, f, g, h) of compound **2** and its metabolites in rat urine.

Fig. S6. Total ion chromatograms (a) and extracted ion chromatograms (b, c, d, e, f, g, h, i, j) of compound **3** and its metabolites in rat urine.

Fig. S7. Total ion chromatograms (a) and extracted ion chromatograms (b, c, d, e, f, g, h) of compound **4** and its metabolites in rat urine.

Fig. S8. Total ion chromatograms (a) and extracted ion chromatograms (b, c, d, e, f, g, h) of compound **5** and its metabolites in rat urine.

Table S1. The effects of **1-5** on cell activity of A549 (human lung adenocarcinoma

cell).

Table S2. The effects of **1-5** on cell activity of HepG2 (human liver cancer cell).

Table S3. The effects of **1-5** on cell activity of HCT116 (Human colorectal cancer cells).

Table S4. The effects of **1-5** on cell activity of MCF7 (Human breast cancer cells).

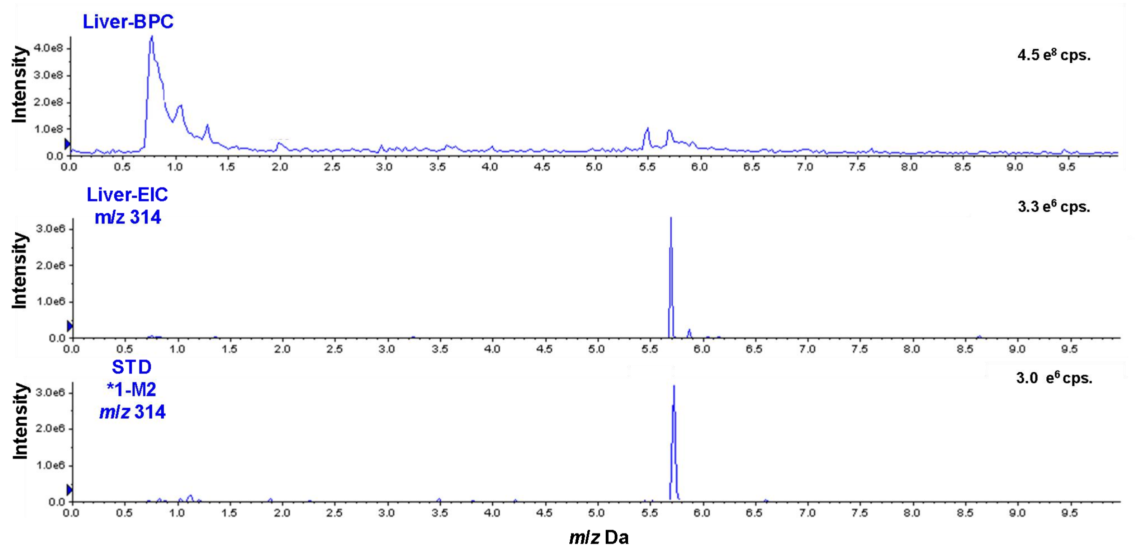

Figure S1. Base peak chromatograms of **1** in rats liver after oral administration (A), extracted ion chromatograms of **1-M2** in rat liver (B), and the standard solution of **1-M2** (C) .

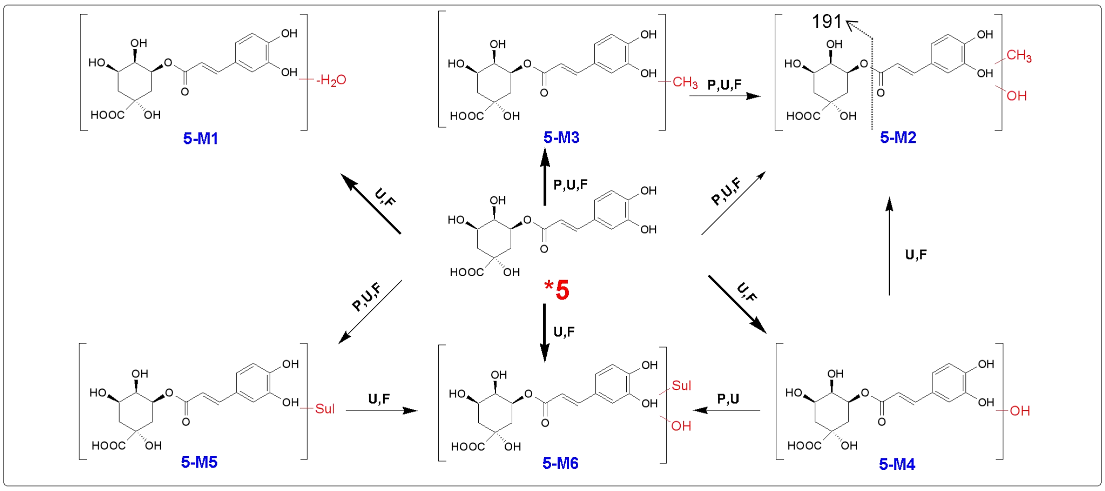

Figure S2. Proposed metabolic pathway for **5** in rats after oral administration.

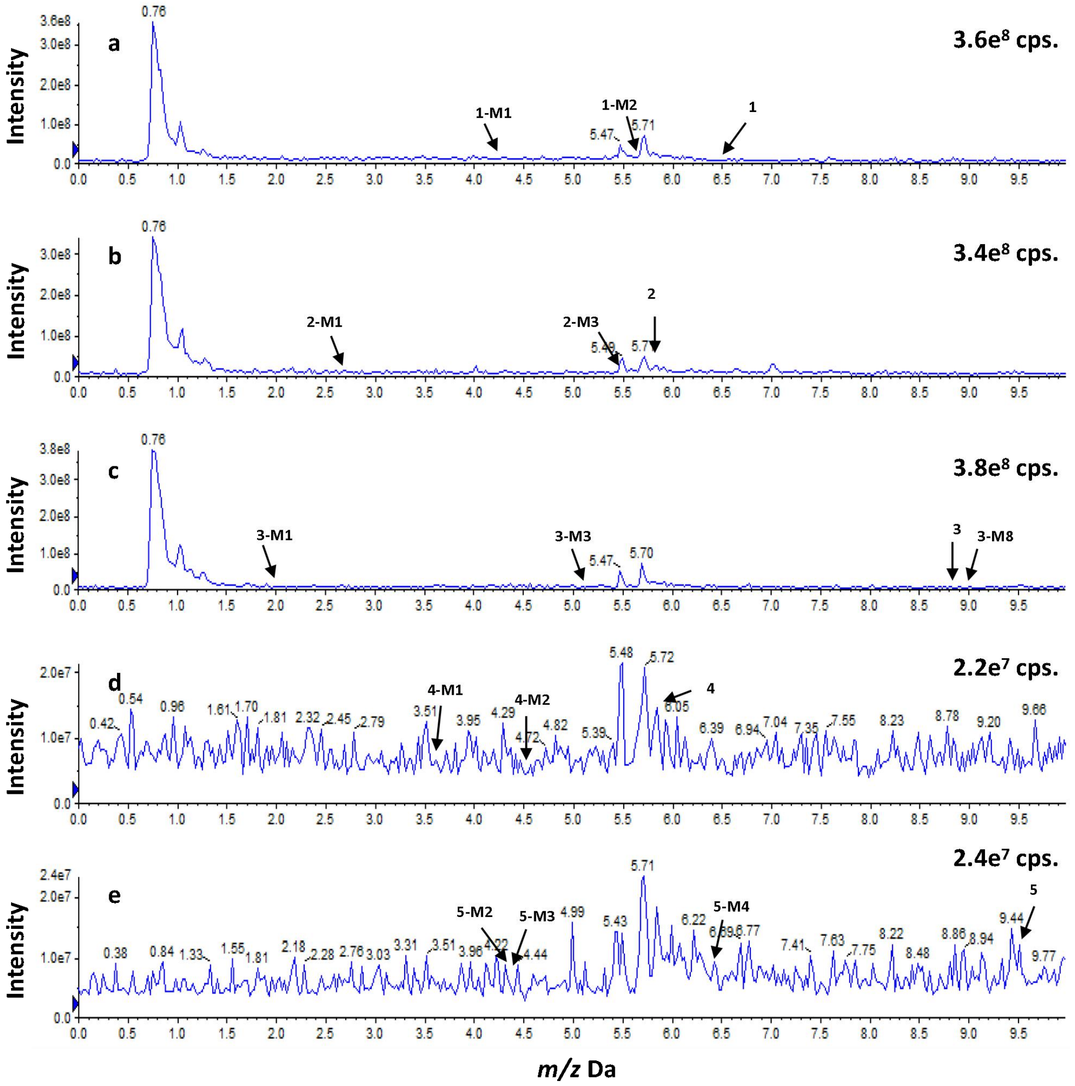

Fig. S3. TIC chromatograms of metabolites of *n*-trans-p-coumaroyltyramine (**1**, a), *n*-trans-p-coumaroyloctopamine (**2**, b), *n*-trans-p-coumaroylnoradrenline (**3**, c), *n*-trans-feruloyloctopamine (**4**, d), phenylpropanoid neochlorogenic acid (**5**, e) in rat liver microsomes in vitro.

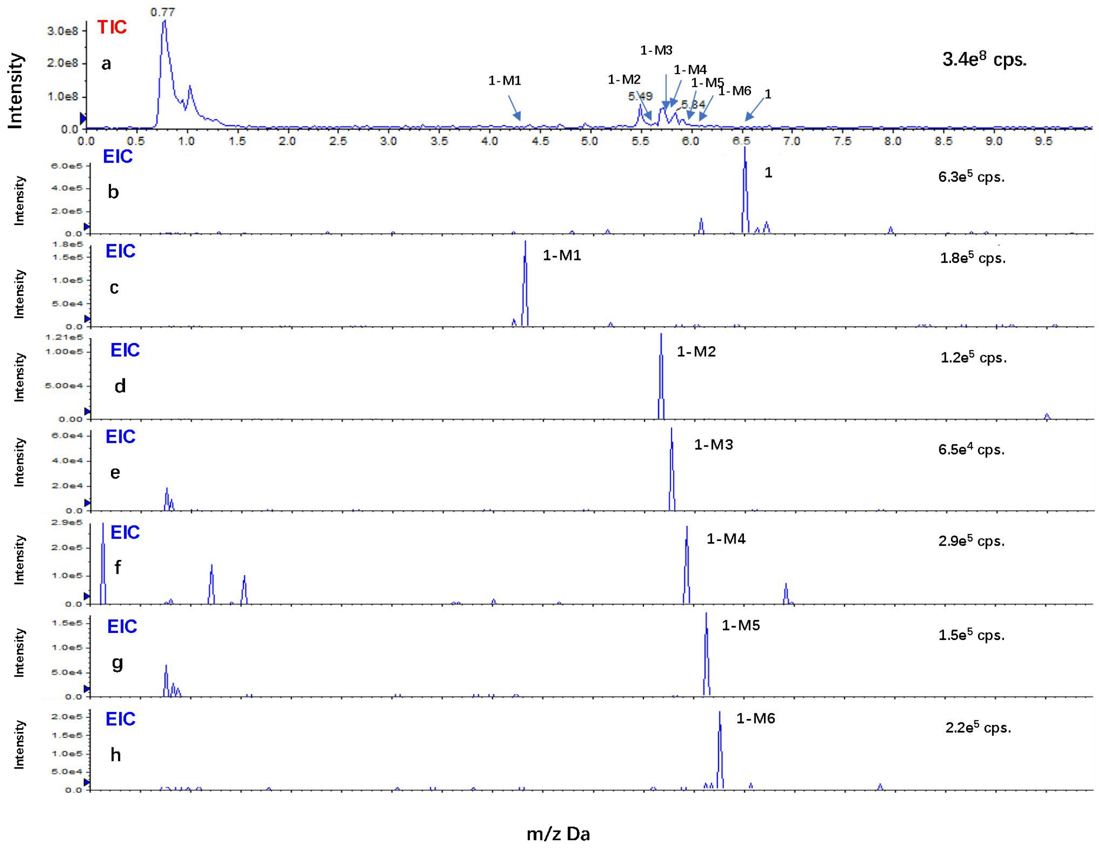

Fig. S4. Total ion chromatograms (a) and extracted ion chromatograms (b, c, d, e, f, g, h) of compound **1** and its metabolites in rat urine.

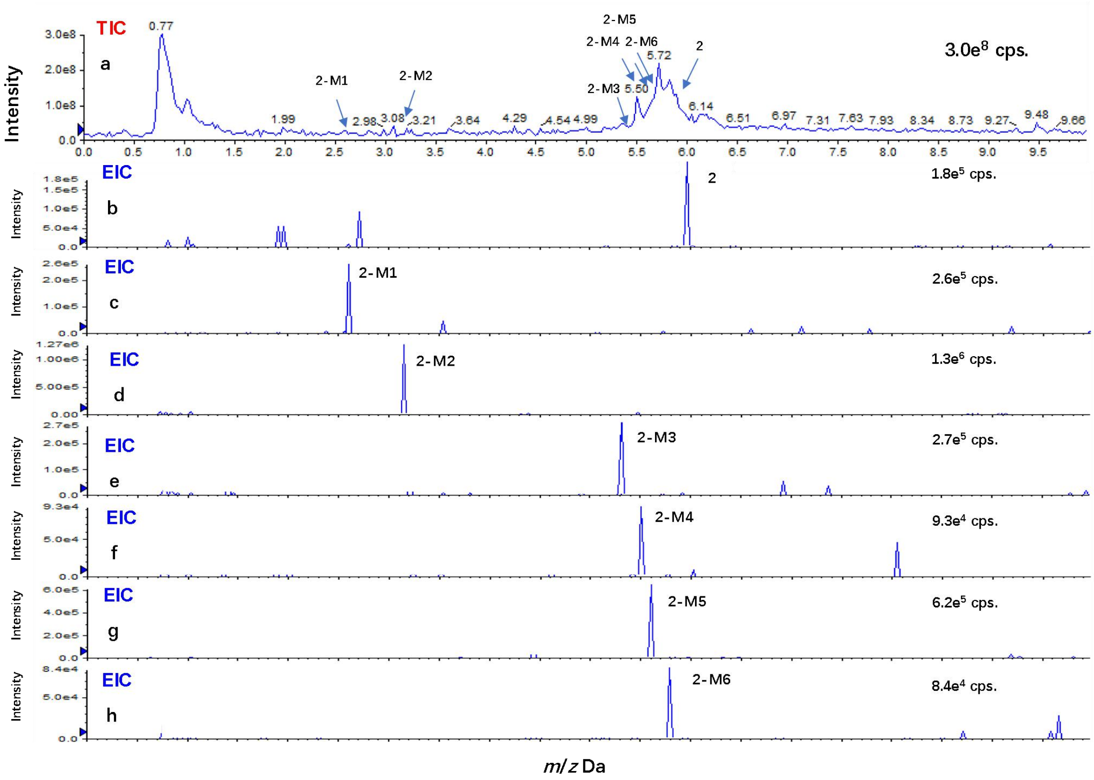

Fig. S5. Total ion chromatograms (a) and extracted ion chromatograms (b, c, d, e, f, g, h) of compound **2** and its metabolites in rat urine.

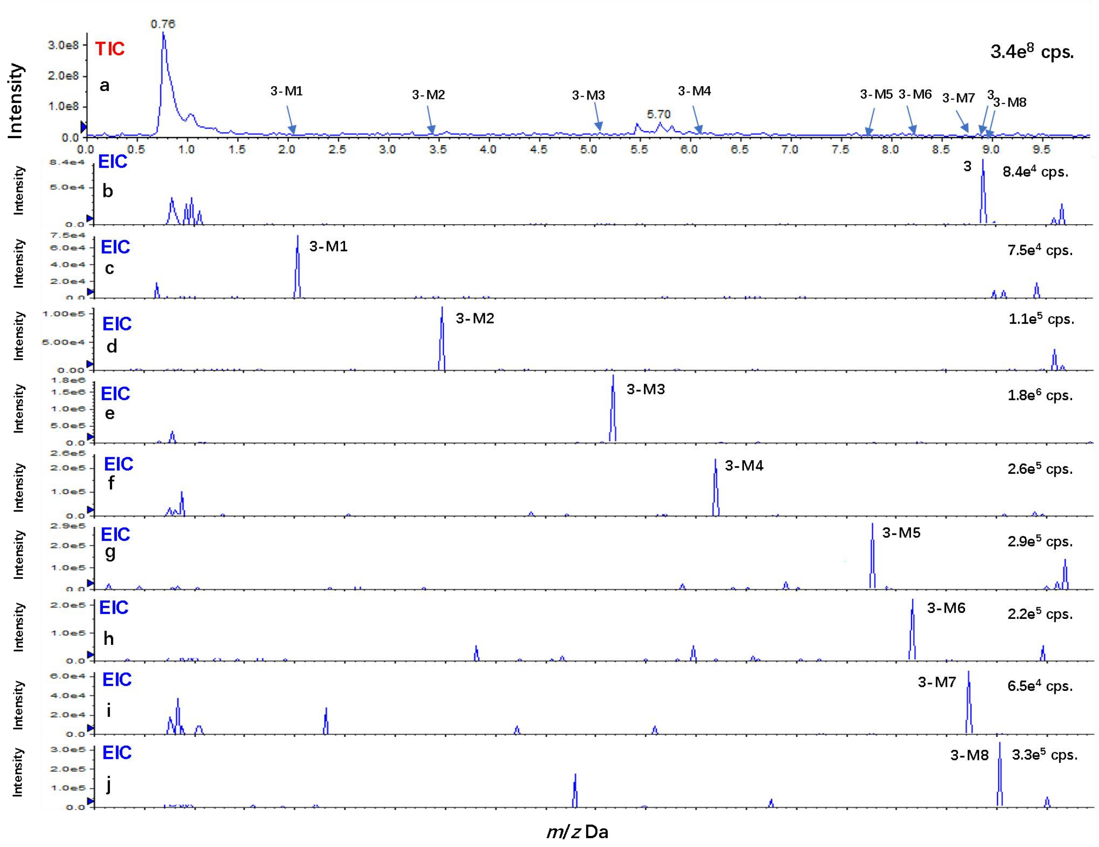

Fig. S6. Total ion chromatograms (a) and extracted ion chromatograms (b, c, d, e, f, g, h, i, j) of compound **3** and its metabolites in rat urine.

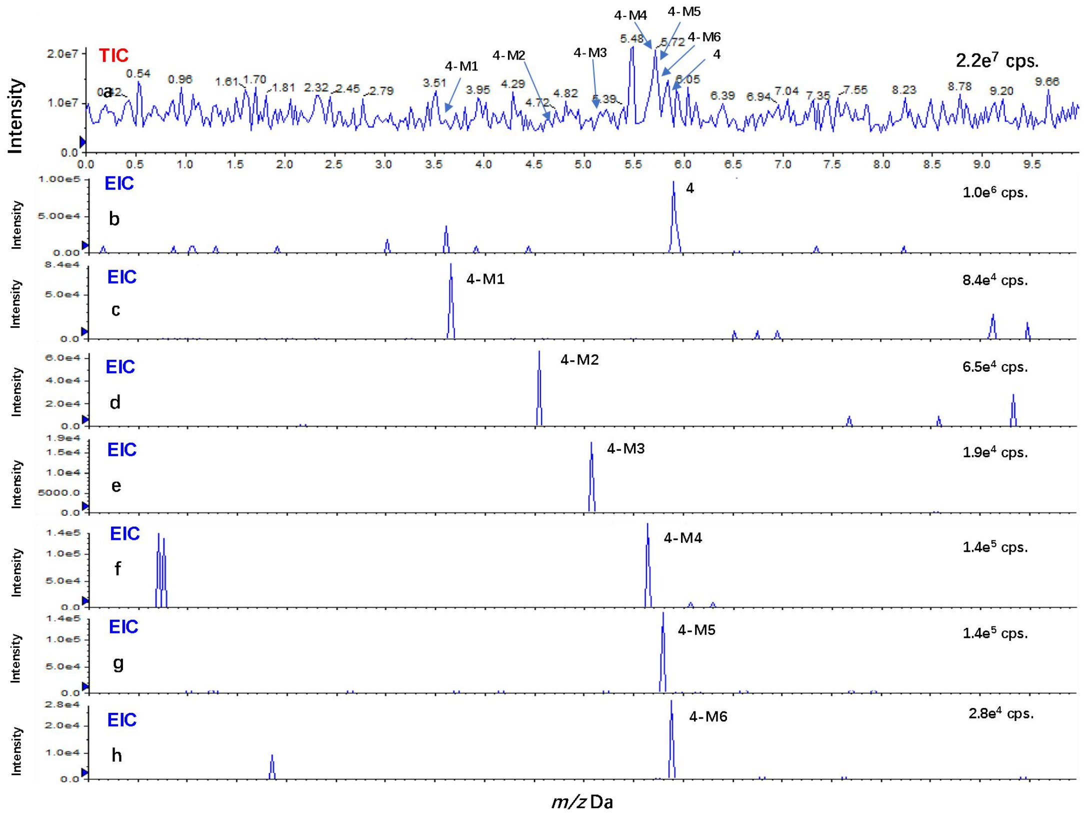

Fig. S7. Total ion chromatograms (a) and extracted ion chromatograms (b, c, d, e, f, g, h) of compound **4** and its metabolites in rat urine.

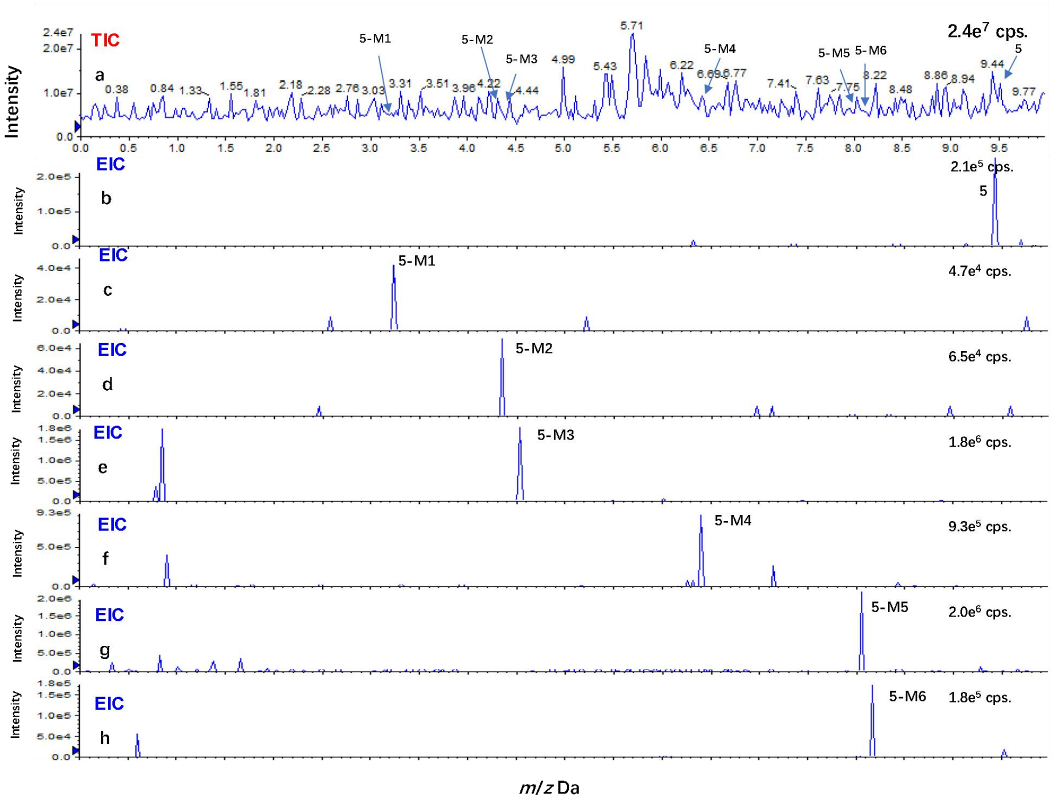

Fig. S8. Total ion chromatograms (a) and extracted ion chromatograms (b, c, d, e, f, g, h) of compound **5** and its metabolites in rat urine.

Table S1. The effects of **1-5** on cell activity of A549 (human lung adenocarcinoma cell).

Group	n	Cell availability（%）		
		24h	48h	72h
Control	9	100±17.80	100±6.93	100±7.28
**1**	9	88.82±7.81	73.61±5.93***	70.39±9.65***
**2**	9	90.91±8.76	78.13±11.78**	90.00±16.00
**3**	9	88.91±6.24	85.44±10.93*	90.50±11.61
**4**	9	86.06±17.06	90.27±9.93*	94.65±9.90
**5**	9	87.26±7.94*	98.61±9.80	87.73±12.73*

Table S2. The effects of **1-5** on cell activity of HepG2 (human liver cancer cell).

Group	n	Cell availability（%）		
		24h	48h	72h
Control	9	100±7.99	100±7.82	100±5.15
**1**	9	153.56±42.55**	162.62±48.48**	139.48±24.10**
**2**	9	139.56±26.88**	164.83±38.21**	139.85±39.56*
**3**	9	126.63±20.89**	125.33±23.30*	114.58±9.79**
**4**	9	116.55±10.34**	102.95±14.74	108.87±6.57*
**5**	9	118.04±21.15*	84.30±8.70**	101.95±5.67

Table S3. The effects of **1-5** on cell activity of HCT116 (Human colorectal cancer cells).

Group	n	Cell availability（%）		
		24h	48h	72h
Control	6	100±10.24	100±14.80	100±3.34
1	6	106.94±23.84	91.26±16.12	60.63±4.64***
2	6	96.46±10.19	95.26±17.11	71.06±8.45***
3	6	87.74±12.40	104.84±6.73	80.48±6.58***
4	6	95.94±6.57	102.56±8.42	100.84±8.35
5	6	80.25±7.51*	85.13±14.86*	47.04±5.07***

Table S4. The effects of **1-5** on cell activity of MCF7 (Human breast cancer cells).

Group	n	Cell availability（%）		
		24h	48h	72h
Control	6	100±7.50	100±8.92	100±6.64
1	6	137.08±5.17**	96.21±17.34	50.85±9.15***
2	6	131.60±14.75**	98.17±13.74	105.30±13.11
3	6	147.71±26.95***	101.12±10.16	96.05±11.35
4	6	200.04±25.17***	99.52±6.19	96.73±6.10
5	6	46.58±8.14***	31.80±5.67***	13.50±2.78***
